# Exploring Medical Artificial Intelligence Readiness Among Future Physicians: Insights From a Medical College in Central India

**DOI:** 10.7759/cureus.76835

**Published:** 2025-01-03

**Authors:** Diwakar Dhurandhar, Mithilesh Dhamande, Shivaleela C, Pooja Bhadoria, Tripti Chandrakar, Jagriti Agrawal

**Affiliations:** 1 Anatomy, Pt. Jawahar Lal Nehru Memorial (JNM) Medical College, Raipur, IND; 2 Prosthodontics, Jawaharlal Nehru Medical College (JNMC), Wardha, IND; 3 Anatomy, Sri Siddhartha Medical College, Tumkur, IND; 4 Anatomy, All India Institute of Medical Sciences, Rishikesh, IND; 5 Community Medicine, Pt. Jawahar Lal Nehru Memorial (JNM) Medical College, Raipur, IND

**Keywords:** ai readiness, artificial intelligence, central india, mairs-ms, medical education

## Abstract

Introduction: Medical students, as future healthcare professionals, are pivotal in the adoption and application of artificial intelligence (AI) in clinical settings. Their ability to effectively engage with AI technologies is shaped by their understanding, attitudes, and perceived significance of AI in medicine. Given the growing prominence of AI in the medical field, it is crucial to evaluate how well-prepared medical students are to integrate and use these technologies proficiently.

Materials and methods: The cross-sectional study was conducted among 482 undergraduate medical students at a medical college in Central India with the objective to evaluate their readiness for the integration of medical AI into their future clinical practice, utilizing the Medical Artificial Intelligence Readiness Scale for Medical Students (MAIRS-MS) questionnaire.

Results: The mean age of respondents was 21.39 ± 1.770 years with 282 (58.5%) male participants. The respondents were almost equally distributed among all Bachelor of Medicine and Bachelor of Surgery (MBBS) batch students. The average MAIRS-MS score came out to be 74.61 ± 10.137 out of a maximum of 110, whereas the mean values of various subscales of MAIRS-MS were as follows: Cognition Factor, 26.23 ± 4.417; Ability Factor, 27.62 ± 4.372; Vision Factor, 10.37 ± 1.803; and Ethics Factor, 10.39 ± 1.789.

Conclusion: Although there is overall readiness for AI among the respondents, significant variation exists among individuals, especially in the areas of Cognition and Ability. The data highlights the necessity for focused educational programs to improve AI knowledge, skills, and ethical understanding, ensuring that every respondent is well-equipped to handle the advancing field of AI in medicine.

## Introduction

Artificial intelligence (AI) is rapidly transforming various fields, including medicine, by introducing innovative tools and methods for diagnosis [1], treatment [2], personalized medicine [3], and patient management [4]. AI technologies, such as machine learning [5], deep learning [6], and natural language processing [7], are progressively being incorporated into medical practice [8], providing improved decision-making capabilities, more accurate diagnostics, and advanced predictive analytics. The integration of AI in healthcare has the potential to revolutionize medical practice, enhancing precision, efficiency, and patient outcomes. However, the successful implementation of AI in clinical settings depends not only on the availability of advanced technologies but also on the readiness and competence of healthcare professionals, particularly future doctors [9].

Medical students, who are the future healthcare providers, play a crucial role in the adoption and utilization of AI in clinical practice [9]. Their readiness to engage with AI technologies is influenced by their knowledge, attitudes, and perceived importance of AI in medicine. As AI continues to gain prominence in the medical field, it is essential to assess the preparedness of medical students to embrace and utilize these technologies effectively.

This study aims to evaluate the readiness of undergraduate medical students in a medical college in Central India to incorporate AI into their future medical practice. The assessment focuses on their awareness, understanding, and attitudes toward AI, as well as the factors that influence their readiness to adopt AI in their clinical work [10]. Understanding the level of preparedness among these students is crucial for developing targeted educational strategies that can equip them with the necessary skills and knowledge to utilize AI effectively in their future careers.

This evaluation is particularly significant in the context of India, where the healthcare system is undergoing rapid changes and the adoption of AI could play a vital role in addressing challenges such as resource constraints and healthcare accessibility [11-14]. By assessing the readiness of medical students, this study seeks to contribute to the broader efforts of integrating AI into the medical curriculum and ensuring that future healthcare professionals are well-prepared to harness the potential of AI in improving patient care.

Thus, the present study was conducted to evaluate the readiness of undergraduate medical students at Pt. Jawahar Lal Nehru Memorial (JNM) Medical College, Raipur, Chhattisgarh, India, for the integration of medical AI into their future clinical practice, utilizing the Medical Artificial Intelligence Readiness Scale for Medical Students (MAIRS-MS) questionnaire [10].

## Materials and methods

Study design

This was a cross-sectional, observational study.

Study center

This study was conducted at Pt. JNM Medical College, Raipur, Chhattisgarh, India.

Sample size

Around 770 undergraduate medical students are presently studying at Pt. JNM Medical College, Raipur. All those who provided informed consent to participate in the study were included via non-probabilistic convenient sampling. 

Inclusion and exclusion criteria

The inclusion and exclusion criteria for sample selection are outlined in Table 1.

**Table 1:** Inclusion and exclusion criteria

S. no.	Criterion type	Criteria
1	Inclusion	Undergraduate medical students across all years of study
2	Inclusion	Students who have provided informed consent to participate in the study
3	Exclusion	Those refusing to participate in the study
4	Exclusion	Incomplete responses

Study tool

The MAIRS-MS scale, developed by Karaca et al. [10], is a reliable and validated tool (Cronbach's alpha = 0.87) designed to assess the readiness of medical students for AI technologies and applications. This scale includes 22 items on a 5-point Likert scale, evaluating students across four key domains: Cognition, Ability, Vision, and Ethics. The Cognition domain assesses students' understanding of AI terminology, logic, and data science. The Ability domain gauges their competence in selecting and using AI applications effectively, as well as their ability to explain these applications to patients. The Vision domain evaluates their capacity to discuss the limitations, strengths, and weaknesses of AI in medicine and to anticipate future opportunities and challenges. Lastly, the Ethics domain measures their adherence to legal and ethical standards when using AI technologies. Each item is scored from 1 to 5, with 1 representing "strongly disagree (SD)" and 5 representing "strongly agree (SA)". The average score for each domain is calculated to determine the overall AI readiness of the respondents. The survey was administered online using Google Forms. Instructions were provided on how to complete the survey, and students were assured of the confidentiality and anonymity of their responses. The survey was open for six weeks to provide sufficient time, enabling a high response rate. The duration of data collection was from July to August 2024. 

Data analysis

Descriptive statistics (mean, standard deviation) were calculated for overall AI readiness and for each dimension of the MAIRS-MS. Unpaired Student's t-test was used to compare the mean MAIRS-MS score on the basis of gender. The mean scores of various subscales were compared with the participants' ages using Pearson's correlation coefficient. IBM SPSS Statistics for Windows, V. 21.0 (IBM Corp., Armonk, NY, USA), was used for the analysis. A p-value of less than 0.05 was considered as significant. 

Ethical considerations

Before the commencement of the survey, all students received an explanation regarding the study's purpose, objectives, and potential benefits. The individuals were afforded the opportunity to decide freely whether they wished to take part. Assurances were given regarding the preservation of anonymity and the confidentiality of data. Furthermore, the study was approved by the Institutional Ethics Committee of Pt. JNM Medical College (approval number: MC/Ethics/2024/503; date: March 8, 2024).

## Results

In the present study, 482 participants responded to the MAIRS-MS questionnaire. The demographic characteristics of the study participants have been tabulated in Table 2. 

**Table 2:** Demographic characteristics of the study participants

Demographic details	Number (n)	Percentage (%)
Gender		
Males	282	58.5
Females	200	41.5
Total	482	
Year of study		
Phase 1	102	21.2
Phase 2	112	23.2
Phase 3 part 1	100	20.7
Phase 3 part 2	89	18.5
Internship	79	16.4
Mean age in years	21.39 ± 1.770	

MAIRS-MS has 22 items and four subscales: Cognition Factor, Ability Factor, Vision Factor, and Ethics Factor. Cognition Factor has eight items. The responses in Likert scale from SD (score of 1) to SA (score of 5) to items of Cognition Factor have been shown in Table 3.

**Table 3:** Responses of participants in Likert scale to items of Cognition Factor of MAIRS-MS SA: strongly agree; A: agree; N: neutral; D: disagree; SD: strongly disagree; AI: artificial intelligence; MAIRS-MS: Medical Artificial Intelligence Readiness Scale for Medical Students

S. no.	Item description	Frequency (percentage)					
		SA	A		N	D	SD
1	I can define the basic concepts of data science	13 (2.7)	170 (35.3)		253 (52.4)	37 (7.7)	09 (1.9)
2	I can define the basic concepts of statistics	17 (3.5)	212 (44.0)		221 (45.8)	25 (5.2)	07 (1.5)
3	I can explain how AI systems are trained	14 (2.9)	142 (29.5)		244 (50.6)	69 (14.3)	13 (2.7)
4	I can define the basic concepts and terminology of AI	13 (2.7)	168 (34.8)		219 (45.4)	74 (15.4)	08 (1.7)
5	I can properly analyze the data obtained by AI in healthcare	19 (3.9)	165 (34.3)		234 (48.5)	52 (10.8)	12 (2.5)
6	I can differentiate the functions and features of AI-related tools and applications	18 (3.7)	160 (33.2)		234 (48.5)	61 (12.7)	09 (1.9)
7	I can organize workflows compatible with AI	17 (3.5)	144 (29.9)		247 (51.3)	68 (14.1)	06 (1.2)
8	I can express the importance of data collection, analysis, evaluation, and safety, for the development of AI in healthcare	30 (6.2)	196 (40.7)	210 (43.6)		43 (8.9)	03 (0.6)

The participants' responses to items of Ability Factor of MAIRS-MS have been tabulated in Table 4.

**Table 4:** Responses of participants in Likert scale to items of Ability Factor of MAIRS-MS SA: strongly agree; A: agree; N: neutral; D: disagree; SD: strongly disagree; AI: artificial intelligence; MAIRS-MS: Medical Artificial Intelligence Readiness Scale for Medical Students

S. no.	Item description	SA	A		N	D	SD
1	I can harness AI-based information combined with my professional knowledge	24 (5.0)	200 (41.5)		219 (45.4)	36 (7.5)	03 (0.6)
2	I can use AI technologies effectively and efficiently in healthcare delivery	31 (6.5)	200 (41.5)		205 (42.5)	41 (8.5)	05 (1.0)
3	I can use AI applications in accordance with its purpose	30 (6.2)	218 (45.2)		200 (41.5)	31 (6.4)	03 (0.6)
4	I can access, evaluate, use, share, and create new knowledge using information and communication technologies	35 (7.3)	213 (44.2)		209 (43.3)	21 (4.4)	04 (0.8)
5	I can explain how AI applications offer a solution to which problem in healthcare	28 (5.8)	182 (37.7)		227 (47.1)	41 (8.6)	04 (0.8)
6	I find it valuable to use AI for education, service, and research purposes	43 (8.9)	238 (49.4)		176 (36.5)	21 (4.4)	04 (0.8)
7	I can explain the AI applications used in healthcare services to the patient	30 (6.2)	179 (37.1)		236 (49.0)	34 (7.1)	03 (0.6)
8	I can choose the proper AI application for the problem encountered in healthcare	18 (3.8)	166 (34.4)	251 (52.1)		43 (8.9)	04 (0.8)

The participants' responses to items of Vision Factor of MAIRS-MS have been tabulated in Table 5.

**Table 5:** Responses of participants in Likert scale to items of Vision Factor of MAIRS-MS SA: strongly agree; A: agree; N: neutral; D: disagree; SD: strongly disagree; AI: artificial intelligence; MAIRS-MS: Medical Artificial Intelligence Readiness Scale for Medical Students

S. no.	Item description	SA	A	N	D	SD
1	I can explain the limitations of AI technology	25 (5.2)	204 (42.3)	210 (43.6)	37 (7.7)	06 (1.2)
2	I can explain the strengths and weaknesses of AI technology	24 (5.0)	214 (44.4)	215 (44.6)	27 (5.6)	02 (0.4)
3	I can foresee the opportunities and threats that AI technology can create	30 (6.2)	199 (41.4)	219 (45.4)	32 (6.6)	02 (0.4)

The participants' responses to items of Ethics Factor of MAIRS-MS have been tabulated in Table 6.

**Table 6:** Responses of participants in Likert scale to items of Ethics Factor of MAIRS-MS SA: strongly agree; A: agree; N: neutral; D: disagree; SD: strongly disagree; AI: artificial intelligence

S. no.	Item description	SA	A	N	D	SD
1	I can use health data in accordance with legal and ethical norms	20 (4.1)	221 (45.9)	215 (44.6)	20 (4.2)	06 (1.2)
2	I can conduct under ethical principles while using AI technologies	17 (3.5)	208 (43.2)	224 (46.5)	28 (5.8)	05 (1.0)
3	I can follow legal regulations regarding the use of AI technologies in healthcare	23 (4.8)	219 (45.4)	213 (44.2)	24 (5.0)	03 (0.6)

The mean scores in each subscale along with the mean MAIRS-MS score of the study cohort are shown in Table 7.

**Table 7:** Mean scores of various subscales and MAIRS-MS along with highest and lowest scores found among the study participants MAIRS-MS: Medical Artificial Intelligence Readiness Scale for Medical Students

	Max score	Mean value	Range	
			Highest score	Lowest score
Cognition Factor	40	26.23 ± 4.417	40	08
Ability Factor	40	27.62 ± 4.372	40	08
Vision Factor	15	10.37 ± 1.803	15	05
Ethics Factor	15	10.39 ± 1.789	15	03
MAIRS-MS	110	74.61 ± 10.137	110	30

**Figure 1:**
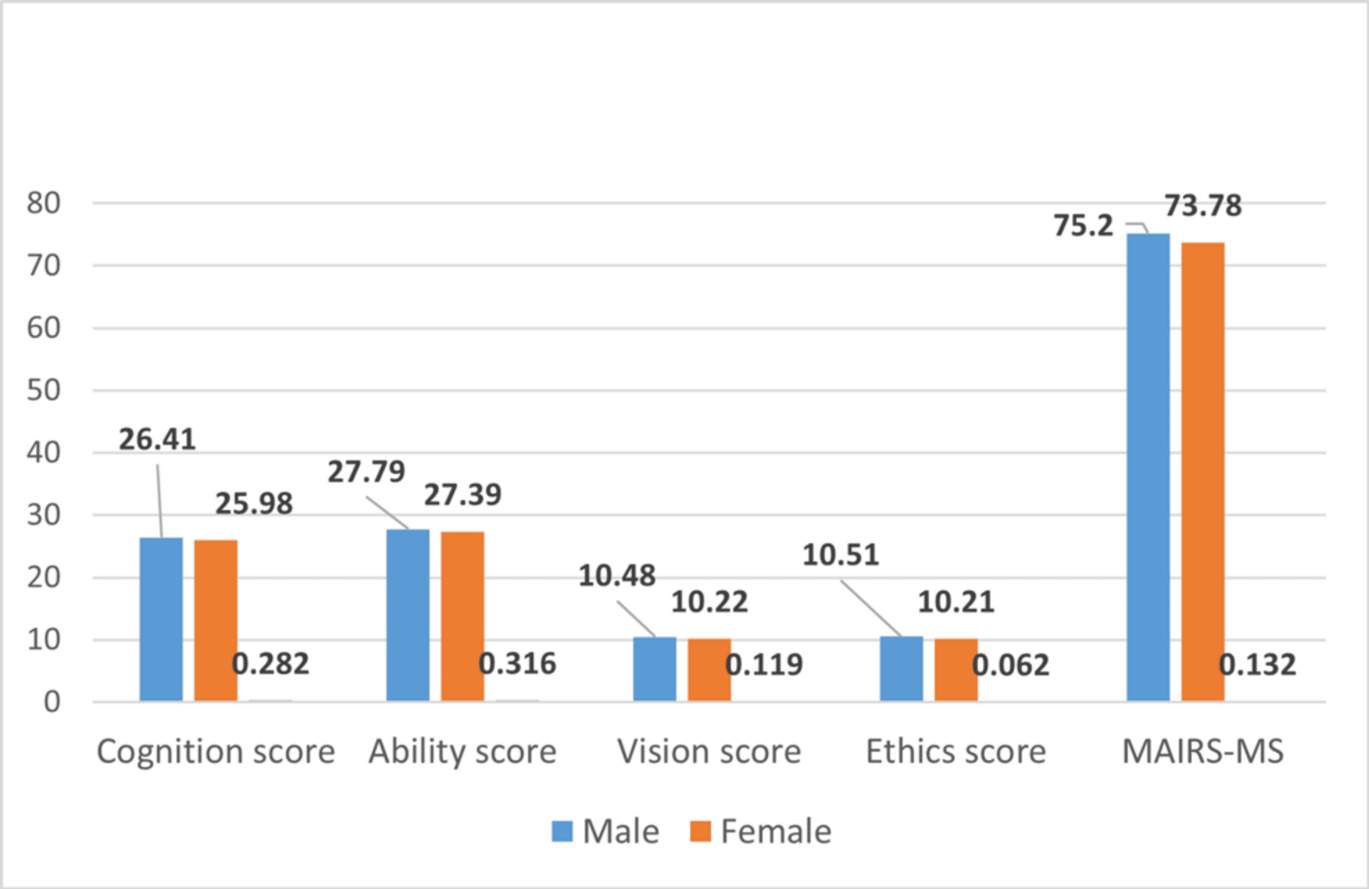
Association of subscale scores and MAIRS-MS scores with gender Various subscale scores and MAIRS-MS scores were compared between male and female genders for any significant difference using the non-parametric Mann-Whitney U test. No significant difference was observed among the two gender groups. MAIRS-MS: Medical Artificial Intelligence Readiness Scale for Medical Students

Figure 1 shows the association of subscale scores and MAIRS-MS scores with gender.

Table 8 shows the correlation between the age of the participants and subscale scores and MAIRS-MS scores.

**Table 8:** Correlation between the age of the participants and subscale scores and MAIRS-MS scores The Ethics subscale score has a negative correlation with age with a p-value of less than 0.05. ^# ^indicates a p-value of less than 0.05, i.e., significant. MAIRS-MS: Medical Artificial Intelligence Readiness Scale for Medical Students

Correlation of readiness score with age	Pearson's correlation coefficient	Two-tailed p-value
Cognition score	-0.12	0.792
Ability score	-0.086	0.060
Vision score	-0.079	0.084
Ethics score	-0.115	0.012^#^
MAIRS-MS	-0.076	0.094

## Discussion

The MAIRS-MS is a comprehensive tool designed to assess the readiness of medical students to engage with AI in their future clinical practice [10]. Developed by Karaca et al. [10], this scale evaluates students across four key domains: Cognition, Ability, Vision, and Ethics. By providing insights into their knowledge, practical skills, perceptions, and ethical considerations related to AI, the MAIRS-MS helps educators and institutions identify areas where additional training and resources may be needed to prepare students for the evolving role of AI in medicine.

In the present study, findings in relation to Cognition Factor indicate that respondents possess a moderate level of understanding regarding AI concepts and applications in the medical field. The diverse range of scores, spanning from 8 to 40, highlights a notable variability in cognitive readiness, with some participants demonstrating a strong command of AI knowledge, while others reveal substantial deficiencies. This study underscores the significant disparity in AI readiness within the participant group, presenting a considerable challenge in designing curricula for such courses. In earlier studies, medical students often reported lower scores in AI knowledge, reflecting limited exposure to AI concepts in their curriculum [15]. Also, a study by Paranjape et al. [16] reported that many students had superficial or no knowledge of AI applications in healthcare. The current score indicates a rise in AI-related knowledge, potentially reflecting greater awareness and growing interest among young people, especially medical students. The current findings show a higher ability score, indicating that students now feel more confident in their ability to work with AI tools. Previous studies, such as one by Pinto Dos Santos et al. [17], revealed that medical students often felt unprepared to apply AI in practice due to a lack of hands-on training. Tung and Dong [15] also found this subscale score to be lower than the present study.

The current score in Vision domain suggests a more positive outlook on AI's future role in medicine. This shift might reflect growing awareness and optimism about AI's transformative potential, as more successful AI applications in healthcare become well-known. This was found in contrary to earlier research, such as the study by Oh et al. [18], which highlighted that many students were uncertain about the future impact of AI in healthcare, with some expressing skepticism about AI's potential benefits.

The current lower score in Ethics Factor in comparison to the findings of Karaca et al. [10] and Tung and Dong [15] may point to challenges in understanding or addressing ethical issues related to AI in healthcare. A primary reason could be knowledge gaps, as many healthcare professionals may lack adequate training or awareness about AI-specific ethical concerns, such as bias, data privacy, and accountability. The complexity of AI ethics, including issues like algorithmic fairness, transparency, and responsibility, can feel overwhelming, leading to lower confidence in ethical preparedness. Another factor could be insufficient institutional support, such as the absence of clear ethical guidelines or lack of training opportunities to handle AI-related dilemmas. A study by Paranjape et al. [16] discusses the need for integrating AI education into medical curricula, highlighting that many students are unprepared for the ethical challenges posed by AI, including issues of bias and privacy. Reddy et al. [19] examined the ethical concerns related to AI in healthcare and emphasized the importance of medical students understanding the implications of AI, particularly regarding accountability and bias. Kolachalama and Garg [9] highlighted the gaps in medical education concerning AI, especially in ethical domains, and calls for more comprehensive discussions on privacy, bias, and accountability. Addressing these challenges through ethics training workshops, clear institutional guidelines, and practical case-based learning can help enhance readiness and bridge these gaps.

When compared to previous studies, the current findings reveal a significant improvement in AI readiness across all domains: Cognition, Ability, Vision, and Ethics. This could be attributed to the rapid integration of AI into everyday life. However, the comparison of this research with older literature, even from just one or two years ago, is influenced by a temporal component, which significantly limits the validity of such comparisons. The MAIRS-MS questionnaire has been widely employed for pre- and post-test evaluations to assess the outcomes of educational interventions related to AI among medical students.

No significant differences in MAIRS-MS scores were observed between genders, reinforcing the argument that gender is not a determinant of AI readiness. Interestingly, a negative correlation was found between the Ethics domain and the age of medical students. The Ethics subscale assesses the knowledge of the ethical and legal frameworks for using AI in medical practice and education. The declining focus on ethics with advancing stages of medical education may stem from students primarily learning from their teachers or senior peers, where ethical considerations often receive less emphasis over time. This highlights the urgent need to integrate ethics in AI as a core component of AI courses when introduced.

The study faced several other limitations as well. It employed convenient non-probabilistic sampling, which typically offers lower internal validity than probabilistic sampling methods. Moreover, confounding variables such as socioeconomic status, digital literacy, and prior experience with technology could have impacted the outcome measures in different ways. Additionally, being a single-center study, its findings would be strengthened by adopting a multicenter approach involving a broader population of medical students, offering more substantial evidence for integrating AI into medical education. MAIRS-MS is utilized to evaluate medical students' perceived readiness for AI. It is an instrument that captures medical students' self-perception of their readiness to integrate AI into their routine medical practice and professional life. This introduces a potential bias. 

A key strength of the study is the lack of existing literature on AI readiness among medical students in India. Additionally, the use of MAIRS-MS, a pre-validated and reliable tool for assessing perceived AI readiness, enhances the validity and scientific rigor of the study's findings.

Designing a curriculum for AI in healthcare for medical students with varying levels of knowledge requires a structured, inclusive, and flexible approach. The curriculum must ensure a balance between inclusivity, foundational learning, and advanced exploration, enabling all students to gain meaningful insights into AI's role in healthcare while addressing the knowledge gaps among them.

## Conclusions

While there is a general readiness for AI among the respondents, notable differences are observed among individuals, particularly in Cognition and Ability. This data underscores the need for targeted educational initiatives to enhance AI knowledge, skills, and ethical comprehension, ensuring that all respondents are adequately prepared to navigate the evolving domain of AI in medicine. This is done by designing and implementing a curriculum which strikes a harmonious balance between inclusivity, foundational knowledge, and advanced exploration, ensuring that all students acquire valuable insights. In the present study, orientation and knowledge regarding the ethical and legal framework for AI in healthcare decreased with age among medical students. To address this challenge, it is crucial to integrate ethical and legal education related to AI throughout the entire medical curriculum, including in clinical phases, with a focus on its relevance and application in real-world healthcare settings.
